# The Role of Vitamin C in Human Immunity and Its Treatment Potential Against COVID-19: A Review Article

**DOI:** 10.7759/cureus.33740

**Published:** 2023-01-13

**Authors:** Austin Moore, Deepesh Khanna

**Affiliations:** 1 Foundational Sciences, Nova Southeastern University (NSU) Florida, Dr. Kiran C. Patel College of Osteopathic Medicine, Clearwater, USA

**Keywords:** vitamin c deficiency, sepsis, covid-19 complications, wound healing and tissue repair, antioxidant, sars-cov-2, coronavirus-19, coronavirus disease, covid-19, vitamin - c

## Abstract

The outbreak of the COVID-19 pandemic has left clinicians around the world searching for viable prevention and treatment options to use against the virus. The important physiologic properties of vitamin C have been well documented regarding its use by immune cells and its role as an antioxidant. It has previously shown potential as a prophylactic and treatment option for other respiratory viruses, and because of this, there has been intrigue into whether these positive outcomes translate into a cost-effective prevention and treatment option for COVID-19. To this point, there have only been a few clinical trials performed to assess the validity of this notion, with very few showing definitive positive outcomes when vitamin C has been incorporated into prophylactic or treatment protocols to use against coronavirus. When being used to specifically treat the severe complications that arise from COVID-19, vitamin C is a reliable option to treat COVID-19-induced sepsis but not pneumonia or acute respiratory distress syndrome (ARDS). As a treatment option, high-dose therapy has shown flashes of promise in a few studies although investigators in these studies often subject the testing group to multimodal therapies that include vitamin C as opposed to just vitamin C alone. Given the role that vitamin C has shown to uphold regarding the human immune response, it is currently advised for all individuals to maintain a normal physiologic range of plasma vitamin C through diet or supplements for adequate prophylactic protection against the virus. More research with definitive outcomes will be needed before it is recommended to provide high-dose vitamin C therapy to prevent or treat COVID-19.

## Introduction and background

Since the start of the COVID-19 pandemic in March 2020, there have been around 658 million confirmed cases of COVID-19, resulting in around 6.68 million deaths worldwide by July 2022 [1]. Severe acute respiratory syndrome coronavirus 2 (SARS-CoV-2), which is the virus responsible for COVID-19, was introduced to humans on December 13, 2019. It was named based on its resemblance to SARS-CoV, which was isolated in February 2003 [2]. The seismic consequences that have resulted from SARS-CoV-2 being introduced to humans have not been seen before with any other iteration of the coronavirus. SARS-CoV-2 causes a myriad of symptoms in those with a mild infection that include cough, fever, fatigue, and gastrointestinal disturbance. The most feared aspect of COVID-19 is the severe complications that are commonly seen in patients with more pronounced infections and preexisting conditions [3-6]. These include pneumonia, acute respiratory distress syndrome (ARDS), septic shock, coagulation dysfunction, liver dysfunction, and acute kidney failure [7]. These complications have resulted in nearly five million hospitalizations in the United States alone as of now [8], with many hospitals experiencing periods of strain during times cases peak.

The search for modalities to combat this overwhelming virus has been underway since the crisis started, and up to this point, there have been many proposed prevention and treatment strategies to supplement the mRNA vaccine being used worldwide to prevent COVID-19 infection. Due to the immense inflammatory response brought about by severe COVID-19 infections, leading eventually to what is known as a *cytokine storm* [9], there has been a search for treatments that address this rise in inflammation while also granting support to immune cells so they may suppress the infection. It has been found that cells of the immune system rely on certain micronutrients to function optimally through different stages in the immune response such as vitamins A, B, C, E, B6, B12, folate, zinc, iron, copper, and selenium [10-12]. Of these micronutrients, vitamin C (in the form of ascorbate, the physiologic form of vitamin C) is the one that has been historically heralded by the public as the most critical micronutrient for immune function.

Current treatment options consist mostly of repurposed drugs such as antivirals (remdesivir, lopinavir, oseltamivir, favipiravir, and ritonavir), immunomodulatory drugs (chloroquine, hydroxychloroquine, and tocilizumab), corticosteroids, angiotensin receptor blockers, and statins [13-15]. Of these options, the only one that has received FDA approval to treat hospitalized patients with COVID-19 is remdesivir [16]. Oral and intravenous vitamin C therapy has a relatively low cost compared to the pharmaceutical drugs that are currently being used as treatments for COVID-19, and if they prove efficacious, they could provide a cost-effective replacement or supplement option to pharmaceutical treatment. The focus of this study will revolve around addressing the validity of the proposed role of vitamin C as a key nutrient required for proper immune function and whether that notion means it can be a viable prevention and treatment modality to use against the unrelenting COVID-19 pandemic.

## Review

Methodology

Literature was identified by searching the terms "COVID-19," "coronavirus," "Vitamin," "Vitamin C," "nutrition," "pneumonia," and "sepsis" in *PubMed*. All the published randomized controlled trials and clinical trials discussed in this study are from 2020 and 2021. Most of the background information was taken from articles published within the last 20 years, with some information coming from articles older than that. Articles were screened for the relevance regarding the topic of this study, and relevant articles were included in the paper as evidence to contribute to the discussion and conclusion portions of this review.

Results

Effects on Immune Cell Function

There has already been a significant amount of research performed over the past few decades to understand the role that vitamin C plays in the human immune response. As it turns out, there are numerous components of the immune system that require vitamin C to function optimally, the micronutrient naturally found in citrus, vegetables, and potatoes [17]. White blood cells, including neutrophils and monocytes, accumulate concentrations of vitamin C up to 100 times greater than that of plasma [10]. This phenomenon by itself indicates this micronutrient is likely required by leukocytes to carry out their immune functions [11]. Vitamin C is a crucial component of both the innate (nonspecific) and adaptive (specific) portions of the immune system [18]. Regarding innate immunity, vitamin C is believed to play a role during the initial chemotactic response of neutrophils shortly after infection [10]. Bozonet et al. showed that following vitamin C supplementation, a 20% increase in neutrophil chemotactic activity was observed [19]. Subsequently, it also contributes to the phagocytosis and killing of microbes by neutrophils once they reach the site of infection [18]. Compromised neutrophils that had decreased chemotactic and phagocytic activity have been isolated from septic patients; this lack of activity is believed to be caused by low levels of vitamin C occurring in high-stress situations [20]. When it comes to the T and B lymphocytes that comprise the adaptive portion of the immune system, vitamin C contributes to their function as well. The maturation, proliferation, and viability of T cells have all been shown to be upregulated by the presence of normal physiologic concentrations of vitamin C [20]. When it comes to B lymphocytes, the cells responsible for generating immunoglobulins (Igs), Vitamin C has been shown to directly affect the number of Igs released from B cells [20]. Prinz et al. demonstrated that supplementation of vitamin C among healthy young adult males showed a significant increase in serum levels of IgA, IgG, and IgM [21]. Cells from both the innate and adaptive immune systems are regulated by cytokines. These molecules are secreted by immune cells themselves to either elicit a pro-inflammatory or anti-inflammatory effect to regulate inflammatory responses [10]. Mikirova et al. performed a study demonstrating the effects of high-dose vitamin C on cytokine levels in cancer patients, finding decreased amounts of the cytokines Interleukin-1 alpha (IL-1 alpha), IL-2, IL-8, and tumor necrosis factor-alpha (TNF-alpha) after high-dose vitamin C infusion [22]. In contrast, Jeng et al. showed that when vitamin C was supplemented with vitamin E in healthy adults, it increased the production of cytokines IL-1 beta and TNF-alpha [23]. Based on these findings, it is likely that vitamin C acts to modulate the levels of cytokines to prevent them from fluctuating in either direction.

Role of Vitamin C as an Antioxidant

In addition to directly affecting immune cells, vitamin C also acts as an important antioxidant to the cells of the immune system. It can help protect cells from both endogenously and exogenously produced reactive oxygen species (ROS) during an immune response [24]. When immune cells respond to an infectious microbe, it has been shown that human leukocytes, neutrophils, in particular, possess the ability to transport the oxidized form of vitamin C across its membrane to use as a defense mechanism against ROS produced during an immune response [25]. Because it is an electron donor, it can directly reduce ROS, including superoxide anions and hydroxyl free radicals that are produced during normal cellular metabolic respiration [26]. Vitamin C also can recover other endogenous antioxidants in the body such as vitamin E and glutathione, returning them to their active state [27]. The oxidative damage of DNA, proteins, and lipids has been correlated to the development of cataracts, cancer, and cardiovascular disease, and vitamin C has been shown to reduce oxidative damage to these molecules [24]. Vitamin C can also indirectly work as an antioxidant through nuclear factor kappa B (NF-kB), which on its own can contribute to the production of ROS [10]. Tan et al. showed that vitamin C can decrease the activation of NF-kB, thereby further decreasing the production of oxidant species in human dendritic cells [28]. In addition to its antioxidant properties, vitamin C also can reduce harmful nitrogen-based compounds such as N-nitrosamines and nitrosamides, both of which are carcinogenic [29].

**Figure 1:**
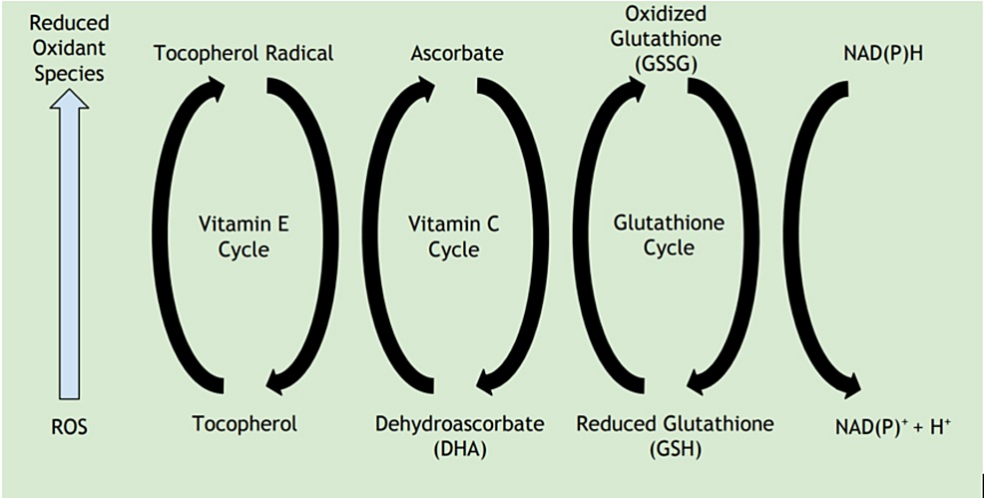
Vitamin C (ascorbate) is shown to reduce both vitamin E and glutathione, two endogenous antioxidants, back to their active states so they may further contribute to the reduction of ROS. Figure credits: Austin Moore. ROS, reactive oxidant species

The question of whether having higher serum vitamin C concentrations provide additional antioxidant benefit has also been explored. Johnston and Cox, who used serum lipid hydroperoxides and Heinz Body quantity in packed red blood cells as a measure of oxidative stress, showed that subjects taking oral vitamin C supplementation saw a 60% to 90% reduction in oxidative stress compared to a placebo control [30]. Pirabbasi et al. performed a study to determine the effectiveness of antioxidant therapy in slowing the progression of chronic obstructive pulmonary disease (COPD) and found that subjects infused with vitamin C alone had a 516% increase in glutathione levels compared to subjects not provided the 500 mg daily supplementation [31]. Glutathione can scavenge free radicals throughout the body, making it arguably the most important component in the antioxidant pathway. Antioxidants are also important in maintaining the integrity and function of endothelial cells. The antioxidant capacity of vitamin C may help to restore this process in subjects who experience compromise to this pathway, such as those with intrahepatic endothelial dysfunction from liver cirrhosis or vascular endothelial dysfunction from chronic smoking [32,33]. Figure 1 shows the pathway to how vitamin C contributes to the reduction of ROS. 

Role in Tissue Repair and Healing

The production of the extracellular matrix during the healing process following damage or infection of tissue also relies on vitamin C [22]. It is responsible for hydroxylating proline and lysine within newly formed collagen, allowing it to mature and stabilize the tissue of a healing wound [34]. Lack of vitamin C leads to this process being compromised. Decreased levels of hydroxylated proline (hydroxyproline) in severe vitamin C deficiency lead to bruising, petechial hemorrhages, and poor wound healing, a condition known as *scurvy* [35].

**Figure 2:**
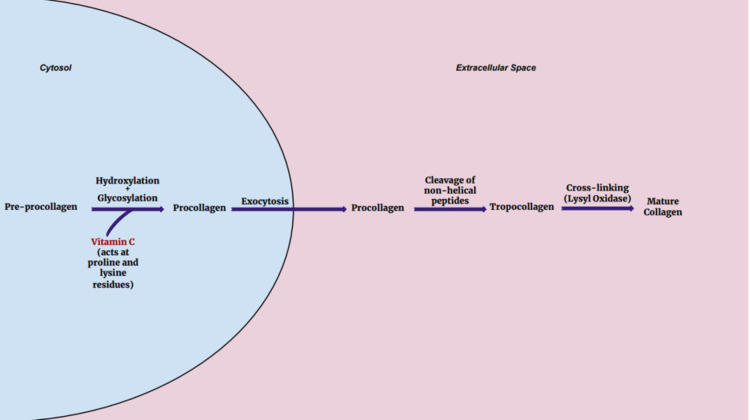
Steps of collagen synthesis displaying the importance of vitamin C early in the synthetic pathway, where it is used by proline and lysine hydroxylases to create procollagen. Figure credits: Austin Moore.

Researchers have been able to demonstrate the critical role vitamin C plays in tissue repair and wound healing through various clinical trials. Gunton et al. sought to determine if oral vitamin C supplementation helped induce healing in patients with chronic foot ulcers. All the patients who daily received 500 mg vitamin C supplementation experienced complete healing of their ulcers, while 44% of the subjects in the control group did not see complete healing [36]. Investigators within the field of oral surgery have also explored the use of vitamin C in assisting with healing following tooth extraction and dental implant procedures. As a result, there is documented evidence suggesting improved soft tissue regeneration following both procedures compared to control groups who did not receive oral vitamin C supplements after the procedure [37,38]. In addition to wound healing, vitamin C/ascorbic acid proves to be beneficial in ameliorating the negative cosmetic effects of aging. The changes in the skin over time are from a combination of the physiologic decline in the integrity and synthesis of collagen as well as ultraviolet light damage from sun exposure. This process is both slowed and improved with regular application of topical vitamin C to sun-exposed areas, with Humbert et al. showing considerable benefit after six months of topical therapy [39]. At the cellular level, vitamin C increases the mRNA levels of type I and type III collagen in the human dermis when skin biopsies were taken from areas on the forearm treated with topical vitamin C compared to skin that was not treated. These samples of treated skin also had higher levels of three posttranslational enzymes that contribute to the synthesis of collagen, including carboxy- and amino-procollagen proteinases and lysyl oxidase [40]. Figure 2 shows the role of vitamin C in collagen synthesis.

Vitamin C for Prophylaxis

Prophylaxis in other respiratory diseases: In the years before the COVID-19 pandemic, there had already been studies performed to determine the effectiveness of vitamin C as a prophylactic treatment for other respiratory infections that share similar symptoms to COVID-19. Studies have demonstrated that those with low levels of vitamin C are at a significantly higher risk of respiratory infection compared to those with normal levels [10,11,18]. The viral cold duration was reduced by about 8% in adults and 13.5% in children using prophylactic daily doses of 200 mg of oral vitamin C before the onset of symptoms in a study done to determine the benefit of daily vitamin C dosing [41]. It has also been made apparent by Hemilä and Chalker that prophylactically supplementing vitamin C decreases the risk of infection with respiratory viruses such as the common cold; however, this was only for individuals undergoing high physical stress [42]. When combined with probiotics, oral vitamin C supplementation showed a 33% decrease in the incidence of respiratory tract infections in preschool-age children [43]. Gorton and Jarvis showed that high-dose oral supplementation of vitamin C managed to prevent or reduce symptoms if taken before or just after the onset of cold- or flu-like symptoms [44]. Unfortunately, not all studies have shown consistent results. Raposo et al. were able to draw an inverse relationship between vitamin C supplementation and the incidence of upper respiratory tract infections (URTIs) for women but not for men [45]. Voß et al. found that vitamin C was shown to prevent respiratory infections when given prophylactically but the results varied depending on the individual subject [46]. There is some evidence to support that prophylactic use of vitamin C helps reduce the severity of respiratory infection symptoms once a subject has already been infected. Wintergerst et al. used 8 g doses of oral vitamin C in patients upon the start of cold symptoms and reported patients saw benefits in both reductions of severity and duration of symptoms [24].

Prophylaxis for COVID-19: After conducting a literature review, it was determined that the topic of whether vitamin C supplementation can be used to prevent COVID-19 infection can be approached from two angles. The first is whether it is beneficial to supplement in subjects who are prone to or are known to have a deficiency, particularly the elderly. Not only the elderly population is most affected by COVID-19 in terms of mortality [47], but it has also been shown that there is a negative correlation between age and serum levels of vitamin C, making the elderly more predisposed to having a vitamin C deficiency [48]. Patients with COVID-19 will likely also experience depletion in serum levels of vitamin C as a direct result of the upregulation of the immune system to combat the infection [49]. Considering it has been shown by studies that vitamin C is essential to human immunity, dietary or pharmaceutical supplementation would inherently reduce the risk of infection while restoring and maintaining overall immune function in these deficient subjects [50].

The second approach now aims to determine whether vitamin C supplementation could benefit subjects who already possess normal physiologic levels. Colunga et al. suggested that oral vitamin C can be combined with oral Quercetin, an antiviral flavonoid, to improve Quercetin’s ability to block viral membrane fusion of SARS-CoV-2, and therefore, it is a safe and inexpensive treatment to prevent infection [51]. Concurrently, it has been suggested that as high doses of 1-2 g/day of oral vitamin C could prevent other upper respiratory infections; this might be a viable option for prophylaxis against COVID-19 infection, but the evidence is lacking [50]. It appears vitamin C supplementation by itself does not provide a striking benefit in preventing COVID-19 infection for those without a deficiency. This was demonstrated by Seet et al. who compared the benefit of oral vitamin C supplementation to other forms of prophylaxis, such as oral hydroxychloroquine and povidone-iodine throat spray. Their study determined that vitamin C treatment alone was inferior to treatment with hydroxychloroquine or povidone-iodine when given prophylactically to healthy adult males to prevent COVID-19 infection [52]. Given its important role in the immune system, some articles propose simply maintaining a well-balanced diet that meets daily recommendations for vitamin C intake so that all components of the immune system can function optimally to adequately combat the SARS-CoV-2 virus in those who have been exposed to it [53,54].

Vitamin C for Treatment of COVID-19 Infection

The amount of data regarding the efficacy of vitamin C as a viable treatment option for those hospitalized from moderate to severe COVID-19 infection has been lacking up until this point, and results from completed studies have been mixed, with few showing definitive results. Hiedra et al. were able to show decreases in inflammatory biomarkers, such as D-dimer and ferritin, in patients who had a 1-g dose of oral vitamin C given every eight hours in addition to standard treatment, but their sample size was only 17 patients and there was no true control group to compare results to said standard treatment alone [55]. In an attempt to improve the invasive mechanical ventilation-free days over 28 days (IMVFD28), Zhang et al. injected 12 g of vitamin C every 12 hours into 56 COVID-19 patients in the ICU to see if their ventilator-free days were increased compared to a placebo. Their study concluded that there was no significant difference in the amount of ventilator-free days but did notice improvements in oxygen saturation and decreased IL-6 levels (a marker of inflammation) in the treatment group compared to the control group [56]. Waqas et al. presented a case report from July 2020 describing a severely ill 74-year-old, COVID-19-positive woman with ARDS and sepsis who, after receiving 11 g/day of IV vitamin C, showed marked improvement in her oxygen saturation and was subsequently extubated three days later [57]. In the aforementioned study that found vitamin C was inferior as a method of prophylaxis compared to povidone-iodine throat spray or oral hydroxychloroquine, subjects that tested positive for COVID-19 upon initial screening were enrolled in another study by Quek et al. to assess interventions to improve Ig production against the virus. They found that oral vitamin C in combination with zinc provided the largest amount of antibody titers 42 days after the discovery of asymptomatic infection compared to other interventions [58]. Arguably, the most promising study to date by Majidi et al. took 100 critically ill hospitalized COVID-19 patients and provided 31 of them with 500 mg of oral vitamin C daily for two weeks while 69 of them remained on standard therapy alone. They concluded that compared to the control group, the intervention group had a higher mean survival duration of eight days compared to four days. There was also a linear relationship between days of vitamin C therapy and survival duration (*B *= 1.66) [59].

Considering these studies showed some positive outcomes, other studies were unable to find any definitive improvement concerning therapy with vitamin C. When adding 6 g/day of IV vitamin C to a treatment regimen consisting of lopinavir/ritonavir and hydroxychloroquine, Jamali et al. were unable to find improvement in oxygen saturation, length of intensive care unit (ICU) stay, and mortality compared to just using the aforementioned treatment alone in hospitalized COVID-19 patients [60]. Li et al. compared outcomes consisting of hospital mortality, daily vasopressor requirement, Sepsis-Related Organ Failure Assessment (SOFA) scores, and ICU length of stay in 8 patients treated with IV vitamin C to 24 patients on standard treatment and were unable to find any significant difference in any of the outcome measures. In this case, the patients who had IV vitamin C therapy had a higher mortality percentage (88% versus 79%) and a higher average SOFA score after treatment (12.4 ± 2.8 versus 8.1 ± 3.5) [61]. In one retrospective cohort study by Al Sulaiman et al., vitamin C was shown to reduce the incidence of thrombotic events in patients receiving enteric vitamin C therapy (1,000 mg once per day), although there was no improvement in in-hospital mortality or 30-day mortality compared to the control group [62].

Vitamin C for Treatment of COVID-19 Complications

Respiratory complications (ARDS and pneumonia): ARDS occurs in around 5% of COVID-19 cases, most of these patients requiring hospitalization and possible invasive intubation [63]. The pathophysiology of ARDS consists of cytokine-induced damage to alveolar and pulmonary capillary membranes which leads to edema in the surrounding lung tissue [64]. The alveolar edema ultimately hinders gas exchange within the lung tissue causing hypoxemia [64]. There has not been much compelling evidence to support that the supplementation of vitamin C has a direct benefit in terms of treating ARDS. Fowler et al. aimed to see if a high-dose vitamin C infusion would benefit patients affected by ARDS, but they were unable to conclude that vitamin C infusion, compared to a placebo, could decrease vascular inflammation and damage in ARDS [65]. It is worth noting that in a sample of 67 COVID-19-positive ICU patients, 82% of them displayed plasma vitamin C levels below 0.4 mg/dL, which is under the normal physiologic range. All of the patients in this study by Tomasa-Irriguible and Bielsa-Berrocal met ARDS criteria according to the Berlin definition [66,67]. Pneumonia is another severe complication of COVID-19 infection, commonly presenting with dry cough, fatigue, dyspnea, and sputum [68]. It has been shown by Mahmoodpoor et al. that in patients with severe pneumonia according to the CURB-65 - confusion, urea, respiratory rate, blood pressure, and 65 years of age or older - criteria, continuous vitamin C infusion at a rate of 60 mg/kg/day for four days decreased the need for mechanical ventilation and vasopressor use but had no significant effect on overall mortality [69].

Sepsis: Along with ARDS, sepsis is another major contributor to mortality in COVID-19 patients who are over 60 years old, have a history of smoking, or have other comorbidities [70]. It has been shown by Marik and Hooper that in septic patients, their vitamin C has readily depleted thanks to a large amount of ROS produced and subsequent antioxidant action of endogenous vitamin C to reduce the number of these ROS [71]. There have been promising findings regarding the treatment of vitamin C to reduce mortality among septic patients. To combat the high rate of turnover of vitamin C, Carr et al. suggested that high-dose IV vitamin C is most effective when treating sepsis as septic patients receiving the normal daily recommendations through diet still showed decreased vitamin C levels [72]. High-dose IV vitamin C treatment has also been shown by Kakodkar et al. to decrease syndecan-1, an endothelial glycocalyx that contributes to mortality in septic patients [70]. When combined with hydrocortisone and thiamine, septic patients treated with 1.5 g of IV vitamin C every six hours showed a distinct decrease in their SOFA scores and none of the patients treated developed organ failure [73]. Fowler et al. were unable to show the benefit regarding the prevention of organ failure for a continuous 96-hour infusion of high-dose vitamin C treatment in septic patients but still claimed it reduced overall mortality [65]. Cardiovascular hypotension is a key factor in the pathophysiology of septic shock for which endogenous vasopressors are produced by the body to combat this phenomenon. Ascorbate (vitamin C) is required by the enzymes dopamine β-hydroxylase and peptidyl glycine α-amidating monooxygenase, which are part of the synthesis of norepinephrine and vasopressin, respectively, two endogenous vasopressors that propose the use for high-dose vitamin C to aid in the treatment of septic shock-induced hypotension [74]. On a more promising note, the treatment of severe sepsis using a high dose (up to 200 mg/kg/day) of IV vitamin C was explored in phase I, a double-blind, randomized, placebo-controlled trial by Fowler et al. [75]. Their findings included a reduction in SOFA scores and decreased vascular injury compared to a placebo control group, all while showing minimal adverse side effects [75].

Discussion

Evidence of the importance of vitamin C regarding immune function is abundant, and the portion of this study that addresses its role is simply a snapshot of the most important functions, with more studies having been performed to further prove that point. It is required for proper leukocyte function in both the innate and adaptive portions of the immune system, controlling the production of pro-/anti-inflammatory cytokines as well as Igs. Vitamin C also has an essential role in helping to reduce the amount of ROS, which, if uncontrolled, can lead to oxidative stress in various locations throughout the body [76]. It is also an important cofactor for enzymatic processes that contribute to tissue repair and healing, with deficiencies in this micronutrient leading to a connective tissue disease known as scurvy.

Prophylactic use of vitamin C may be used to prevent non-COVID-19 respiratory infections and is particularly useful in those who have a vitamin C deficiency. Maintaining a daily intake of 75 and 100 mg for men and women, respectively, as recommended by the U.S. Institute of Medicine [77], is the simplest prophylactic strategy to ensure adequate protection from most respiratory infections. Supplementation to reach a higher daily intake may also be used in individuals without a deficiency if they have had exposure or feel they are at risk for contracting a viral respiratory infection, although the amount of evidence to support this strategy is limited. High doses in nondeficient individuals may also be used to prevent the progression of the infection once symptoms have started.

In terms of prophylactic use against COVID-19, vitamin C supplementation could be deployed as a strategy to minimize the chance of infection in individuals who possess low levels of physiologic vitamin C, particularly in elderly patients. This notion is based on the data to support the importance of vitamin C for overall immune function and that being deficient inherently would increase one’s risk of infection. For those who do not have a deficiency, there is a lack of evidence that supplementing vitamin C would prevent COVID-19 infection and further studies would need to be performed for a compelling case to be made that it is a feasible prophylactic option.

As a treatment option for those already infected with COVID-19, the number of clinical trials to decipher whether vitamin C is an efficacious treatment option has been small. Of the evidence available, no study has been able to definitively conclude that vitamin C may be used to alleviate the symptoms of coronavirus infection and only some were able to show improvement in their desired outcome measures. Even if a study was able to show improvement in some of their outcome measures (such as inflammatory biomarkers and oxygen saturation [55,56]), it could not conclude that vitamin C provided a significant difference for other very important outcome measures such as overall mortality [56,60-62]. The aim of this study then turned to see if vitamin C has had any success in treating some of the specific complications that arise from severe coronavirus infections. Sepsis, being one of the more common contributors to mortality in severe COVID-19, has been shown to be improved with the use of vitamin C thanks to the micronutrient’s immune-supportive and antioxidant properties, although the same benefit has not yet been observed with vitamin C treatment of ARDS or pneumonia, the two most common respiratory complications of severe COVID-19.

## Conclusions

At this time, the basis for using vitamin C to prevent and/or treat COVID-19 infection has not been well defined based on the clinical research that has been performed. It does have a defined role as a necessary micronutrient for leukocyte function and antioxidant activity, this being the only foundation as to why it should be used as a COVID-19 treatment option with little to no definitive clinical evidence to support this notion. Evidence has been presented that shows vitamin C has had some success in preventing other respiratory infections as well as treating some severe COVID-19 complications, but further studies are warranted to show that it is an efficacious treatment option. Currently, it is recommended to maintain an adequate daily intake of vitamin C through sources such as citrus, vegetables, and potatoes or through vitamin C supplementation to ensure the immune system is functioning optimally in case of exposure or contraction of the coronavirus. High-dose treatment for moderate-to-severe infection has not yet shown to be an effective lone treatment option and should only be used along with more successful options to date such as corticosteroid, antibody, and antiviral therapy. Hopefully, given the safety and cost-effectiveness of vitamin C therapy, future studies will provide more definitive evidence to show it is a viable treatment option as it is likely the world’s battle with this virus will continue for years to come.
